# Physical Association of *Saccharomyces cerevisiae* Polo-like Kinase Cdc5 with Chromosomal Cohesin Facilitates DNA Damage Response*

**DOI:** 10.1074/jbc.M116.727438

**Published:** 2016-06-20

**Authors:** Sujiraporn Pakchuen, Mai Ishibashi, Emi Takakusagi, Katsuhiko Shirahige, Takashi Sutani

**Affiliations:** From the ‡ Research Center for Epigenetic Disease, Institute of Molecular and Cellular Biosciences, University of Tokyo, Tokyo 113-0032 and; the § Graduate School of Bioscience and Biotechnology, Tokyo Institute of Technology, Yokohama, Kanagawa 226-8503, Japan

**Keywords:** ChIP-sequencing (ChIP-seq), chromosomes, DNA damage response, Saccharomyces cerevisiae, serine/threonine protein kinase, polo-like kinase, cohesin, priming phosphorylation

## Abstract

At the onset of anaphase, a protease called separase breaks the link between sister chromatids by cleaving the cohesin subunit Scc1. This irreversible step in the cell cycle is promoted by degradation of the separase inhibitor, securin, and polo-like kinase (Plk) 1-dependent phosphorylation of the Scc1 subunit. Plk could recognize substrates through interaction between its phosphopeptide interaction domain, the polo-box domain, and a phosphorylated priming site in the substrate, which has been generated by a priming kinase beforehand. However, the physiological relevance of this targeting mechanism remains to be addressed for many of the Plk1 substrates. Here, we show that budding yeast Plk1, Cdc5, is pre-deposited onto cohesin engaged in cohesion on chromosome arms in G_2_/M phase cells. The Cdc5-cohesin association is mediated by direct interaction between the polo-box domain of Cdc5 and Scc1 phosphorylated at multiple sites in its middle region. Alanine substitutions of the possible priming phosphorylation sites (*scc1-15A*) impair Cdc5 association with chromosomal cohesin, but they make only a moderate impact on mitotic cell growth even in securin-deleted cells (*pds1*Δ), where Scc1 phosphorylation by Cdc5 is indispensable. The same *scc1-15A pds1*Δ double mutant, however, exhibits marked sensitivity to the DNA-damaging agent phleomycin, suggesting that the priming phosphorylation of Scc1 poses an additional layer of regulation that enables yeast cells to adapt to genotoxic environments.

## Introduction

The polo-like kinases (Plks)^2^ are a conserved subfamily of serine/threonine (Ser/Thr) protein kinases and are important regulators in diverse aspects of the cell cycle and cell proliferation (1–4). Among the four mammalian Plks, Plk1 is the most extensively studied enzyme, and it has been shown to regulate various cellular and biochemical events in M phase, including centrosome maturation, bipolar spindle formation, mitotic entry, activation of anaphase-promoting complex, and cytokinesis. In the budding yeast *Saccharomyces cerevisiae*, Cdc5 is an only apparent Plk homologue, plays multiple roles in mitotic progression, and thus appears to be functionally relevant to Plk1. Consistent with its diverse roles, Plk1/Cdc5 phosphorylates various substrates in the cell (1, 2, 5, 6), and the full range of the substrates remains to be identified.

One of the characteristic features of the Plks is the presence of the polo-box domain (PBD) in the C-terminal non-catalytic region. PBD functions in targeting the catalytic activity of Plk to its substrate proteins at specific subcellular sites (7). Biochemical and structural studies have revealed that PBD is a phosphopeptide binding module and preferentially binds to a peptide motif consisting of a phosphoserine/phosphothreonine preceded by a Ser residue (S-(Ser(P)/Thr(P)) (8–10). Studies on several Plk1 substrates, including Cdc25C and Grasp65, have led to a widely accepted model, in which PBD first binds to a site on a protein that has been previously phosphorylated by a mitotic priming kinase like Cdk (cell cycle-dependent kinase) and promotes phosphorylation of the same protein at another site by Plk1 (1, 2). PBD of Plk, as well as priming phosphorylation in substrates, is thus believed to play a critical role in specifying the target and timing of Plk-driven phosphorylation. However, a recent study using a yeast *cdc5* mutant defective in PBD-dependent substrate targeting has shown that the phosphopeptide binding mediated by PBD is not essential for mitotic progression and cell viability but for maintenance of spindle pole body integrity (11). Therefore, Plk1 may target a subset of its substrates through a PBD-independent mechanism. It remains to be explored for each Plk1 substrate whether it is phosphorylated by a priming kinase and, if so, how physiologically relevant the priming phosphorylation is.

The cohesin complex is one of the key players in chromosome segregation and is an important target of mitotic regulations (12). It is a ring-shaped protein complex and is thought to embrace two sister chromatids produced by DNA replication, thereby preventing them from falling apart in G_2_ phase. This sister chromatid cohesion is essential for successful chromosome segregation, because it allows the two kinetochores on the sister chromatids to be attached to opposite spindle poles. In mitotic anaphase, proteolytic cleavage of a cohesin subunit Rad21 (Scc1 or Mcd1 in yeast) by a protease separase (Esp1 in yeast) liberates sister chromatids from cohesion and allows chromosome segregation to begin (13–15). The cleavage of Rad21 by separase is therefore a crucial and irreversible step in mitosis and is tightly regulated by multiple mechanisms. One of the important factors in these regulations is a separase-binding protein, securin (Pds1 in yeast). Securin inhibits separase proteolytic activity in metaphase cells. Upon anaphase initiation, the activated anaphase promoting complex induces degradation of securin through ubiquitination, which results in rapid activation of separase (16–18).

Besides securin, Plk1/Cdc5 also regulates behavior of the cohesin complex in mitotic cells through two different mechanisms. First, Plk1 promotes dissociation of the majority of cohesin complexes from chromosome arms in prophase, leaving a small fraction of cohesin tethering sister chromatids until anaphase onset. This so-called “prophase pathway” is observed in vertebrates, independent of separase activity, and is induced by Plk1-driven phosphorylation of, at least, cohesin subunit SA2 (19, 20). Second, Plk1/Cdc5 increases cohesin's susceptibility to separase protease. In budding yeast, phosphorylation of cohesin complex by Cdc5 enhances the rate of cleavage by Esp1/separase both *in vitro* and *in vivo* (21, 22). Rec8, a meiotic isoform of Scc1, is similarly phosphorylated by Cdc5 for its timely cleavage in meiosis I (23, 24). In humans, *in vitro* cleavage reaction of Rad21 subunit by separase is also accelerated by Plk1-dependent phosphorylation of Rad21 (20). In both yeast and human, Plk1/Cdc5 phosphorylates Rad21/Scc1 at several sites, and among them, two (Ser-175 and Ser-263; residue numbers are in budding yeast Scc1) are located near the separase cleavage sites (Arg-180 and Arg-268). Alanine substitution of these two phosphorylation sites (S175A and S263A) impairs Scc1 cleavage and chromosome segregation almost completely in yeast anaphase cells when combined with securin gene deletion (*pds1*Δ) (21), which presumably results in reduced separase activity because binding to securin is prerequisite for full activation of separase in anaphase after securin degradation (25, 26). Hence, Pds1/securin and Scc1 phosphorylation by Cdc5/Plk1 are believed to function redundantly in efficient and timely cleavage of Scc1 at anaphase onset.

In this study, we discovered using chromatin immunoprecipitation (ChIP) analysis that budding yeast polo-like kinase Cdc5 was associated with cohesin complex that is bound to mitotic chromosome arms in a PBD-dependent manner. In cohesin-depleted cells, Cdc5 binding to chromosomes was almost completely abolished, revealing that among chromosome-bound proteins, cohesin is the only major target of PBD-dependent Cdc5 binding. Biochemical analysis unveiled that Cdc5 recognized a middle region of cohesin subunit Scc1 that multiple Ser/Thr residues in this region (besides Ser-175 and Ser-263, the target sites of Cdc5 itself) were phosphorylated in mitosis and that the phosphorylation is required for PBD recruitment. Preventing the phosphorylation by alanine substitutions of these Ser/Thr residues (*scc1-15A*) impaired yeast cell growth only moderately when combined with *pds1* gene deletion. Interestingly, the same double mutant (*scc1-15A pds1*Δ) revealed marked sensitivity to the DNA-damaging agent phleomycin, indicating that the priming phosphorylation of Scc1 plays a more significant role under specific circumstances.

## Results

### 

#### 

##### Cdc5 Is Co-localized with Cohesin on Yeast Chromosomes

**FIGURE 1.**
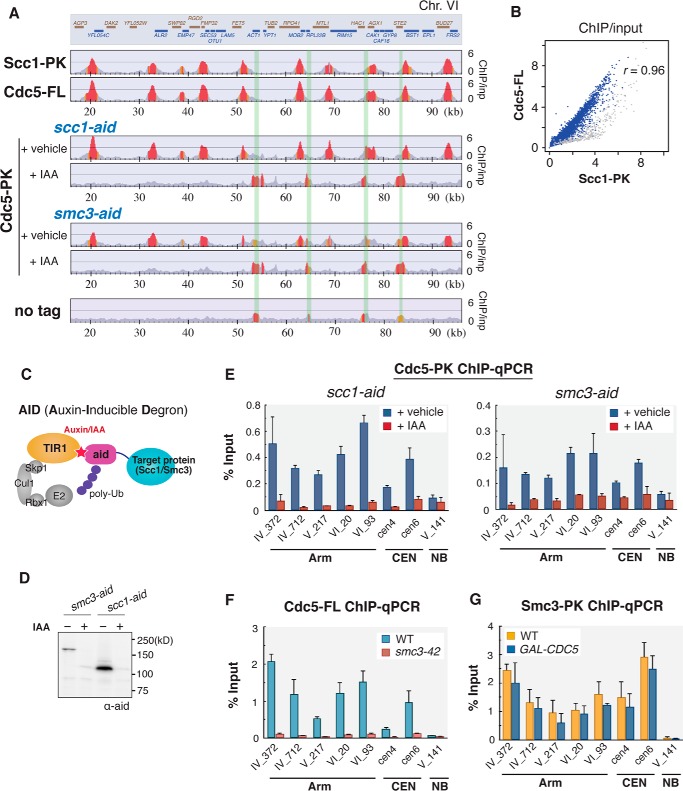
**Co-localization of budding yeast polo-like kinase Cdc5 and cohesin on mitotic chromosome arms.** *A,* ChIP-seq profiles of a PK-tagged cohesin subunit Scc1 (Scc1-PK) and FLAG-tagged Cdc5 (Cdc5-FL) across an 80-kb region (16–96 kb) on *S. cerevisiae* chromosome VI (*Chr. VI*). The *y* axis represents a fold enrichment ratio, or ChIP/input value (60), and reflects the probability at which each protein is bound to the corresponding genome site. The Scc1-PK Cdc5-FL double-tagged cells were arrested at G_2_/M phase by benomyl and subjected to anti-PK and anti-FLAG ChIP-seq analyses. To reveal dependence of Cdc5 binding on cohesin, either of the cohesin subunit Scc1 or Smc3 was specifically depleted by aid system (*scc1-aid* or *smc3-aid*). Addition of IAA (+*IAA*) induced Scc1 or Smc3 subunit degradation. Vehicle-treated cells (+*vehicle*) were used as controls. The peaks highlighted in *red* and *orange* indicate statistically significant enrichment with ChIP/input values of more than 2 and 1.5, respectively. Regions shaded in *green* correspond to peak sites in control ChIP-seq, where cells with no epitope tag (no tag) were subjected to ChIP-seq analysis and represent hyper-chippable regions (30). The *top box* depicts the position of open reading frames (ORFs). *Brown* and *blue bars* represent transcripts on Watson and Crick strands, respectively. *B*, genome-wide correlation between Scc1 and Cdc5 ChIP-seq results. Cdc5-FL and Scc1-PK ChIP-seq ChIP/input value values at each 1-kb genome bin were plotted. Regions surrounding the centromeres (±10 kb) are shown in *gray*. Cdc5-FL and Scc1-PK ChIP/input values showed strong correlation (Pearson's correlation, *r*, of 0.96) along the chromosome arms. *C,* schematic picture of the aid system used to deplete cohesin subunit, Scc1 or Smc3. Auxin (or its derivative IAA) promotes binding of *aid* module to TIR1, which results in poly-ubiquitination and subsequent degradation of the aid-fused target protein. *D,* verification of cohesin subunit depletion by *aid* system. Smc3 or Scc1 protein fused with the *aid* module was detected by anti-aid Western blotting. +, IAA-treated; −, vehicle-treated. *E,* quantification of Cdc5 binding in *scc1-aid or smc3-aid* strain by ChIP-qPCR. The used qPCR loci correspond to cohesin localization sites on chromosome arms (*Arm*) or at the centromeres (*CEN*), except the no binding (*NB*) site where no cohesin accumulation was seen in ChIP-seq profiles. *F,* Cdc5-FL ChIP-qPCR analysis in cohesin temperature-sensitive mutant, *smc3-42*. Wild-type (*WT*) and *smc3-42* strains possessing FLAG epitope-tagged *CDC5* gene were cultured at 23 °C and arrested in G_1_ phase by α-factor. To inactivate cohesin, cells were shifted to restrictive temperature (35 °C) for 30 min while arresting at G_1_. Then, the cells were released into benomyl-containing media at 35 °C for 2 h. The resultant G_2_/M phase cells were subjected to ChIP-qPCR analysis. *G,* Smc3-PK ChIP-qPCR analysis in Cdc5-depleted cells. Cells of *SMC3-*PK (WT) or *SMC3-*PK P*GAL-CDC5* (*GAL-CDC5*), where Cdc5 is expressed from galactose-dependent promoter, were grown in galactose-containing media and arrested in G_1_ phase by α-factor. The cells were subsequently cultured in galactose-free YPD media for 30 min to repress Cdc5 expression and then released from the arrest and re-arrested in G_2_/M phase by cultivating in YPD with benomyl for 2 h. Chromosomal binding of Smc3 in the resultant cells was measured by ChIP-qPCR. The qPCR locus name on chromosome arms represents chromosome number (*roman numerals*) and coordinate (*arabic numerals* following “_,” in kb). *Error bars* indicate standard deviations (*n* = 2, technical replications in qPCR measurements).

ChIP-chip (ChIP on DNA chip) analysis of budding yeast Cdc5 revealed that its genome-wide distribution resembled that of cohesin, as we reported previously (27). To understand the physiological significance of the observed co-localization, we first re-analyzed the distribution profile of Cdc5 along the yeast genome, using ChIP-seq (ChIP followed by DNA sequencing) technique, which produces less noisy and quantitatively more accurate data than ChIP-chip. Yeast cells containing the FLAG-tagged *CDC5* (Cdc5-FL) gene were arrested at G_2_/M phase by the microtubule-destabilizing reagent benomyl, fixed, and lysed. Then DNA fragments bound to Cdc5 protein were immunopurified, and the obtained DNA was sequenced by a massively parallel DNA sequencing instrument. The resultant ChIP-seq profile indicates that Cdc5 is located mostly at transcriptional convergent regions, which are characteristic of budding yeast cohesin-binding sites (Fig. 1*A*) (28). Indeed, comparison of ChIP-seq profiles of Cdc5 and a cohesin subunit Scc1 clearly indicates co-localization of these proteins along chromosome arms (Fig. 1*A*). Correlation plot of ChIP-seq signal intensity reveals that the co-localization was observed universally along chromosome arms (Pearson's correlation = 0.96; Fig. 1*B*). A notable exception is pericentromeric regions, where cohesin is highly enriched, but Cdc5 binding was less pronounced (*gray dots* in Fig. 1*B*).

We then examined whether Cdc5 binding to the cohesin localization sites is dependent on the integrity of the cohesin complex. Scc1 or Smc3, a subunit of cohesin, was C-terminally fused to the aid (auxin-inducible degron) module, and selective degradation of the fused protein was induced by addition of an auxin derivative, indole-3-acetic acid (IAA) (Fig. 1*C*) (29). Depletion of Scc1 and Smc3 proteins was verified by Western blot analysis (Fig. 1*D*). ChIP-seq analysis of Cdc5 in these cohesin-depleted cells revealed the disappearance of Cdc5 from the cohesin-binding sites. The peaks newly emerged in the cohesin-depleted cells were most likely to be a false-positive signal at “hyper-chippable” regions, because these peaks were observed also in cells without epitope tagging (shaded in *green* in Fig. 1*A*) (30), and were independent of Cdc5. The reduced Cdc5 binding to chromosomes in *scc1-aid* and *smc3-aid* strains was also validated by quantitative PCR (qPCR) analysis at multiple genomic loci of DNA obtained in Cdc5-PK ChIP (Fig. 1*E*). Although Cdc5-PK was highly enriched at representative cohesin sites along the chromosome arms (*i.e.* 372 and 712 kb of chromosome IV, 217 kb of chromosome V, and 20 and 93 kb of chromosome VI) in cells with an intact cohesin (+ vehicle), it was dissociated from these sites upon cohesin depletion (+ IAA). Consistent with these results, Cdc5 dissociation from the cohesin-binding sites was also observed in ChIP-qPCR analysis in temperature-sensitive cohesin mutant (*smc3-42*; Fig. 1*F*). In contrast, we observed that chromosome binding of cohesin complex was not affected in Cdc5-depleted cells where expression of Cdc5 was shut off (Fig. 1*G*). This result is consistent with the notion that unlike vertebrates, Plk1-dependent cohesin dissociation from prophase chromosomes is not seen in budding yeast. Taken together, we conclude that Cdc5 protein is associated with the cohesin complex that is bound to G_2_/M phase chromosome arms.

##### Cdc5 Binding to Chromosomes Is Dependent on the Polo-box Domain

**FIGURE 2.**
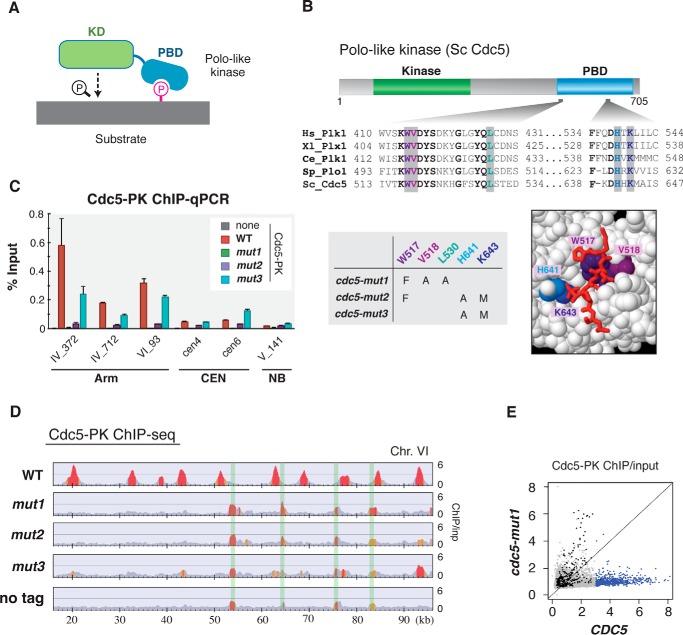
**PBD of Cdc5 is required for its co-localization with cohesin.** *A,* schematic view of PBD-dependent substrate recognition of polo-like kinase. A polo-like kinase possesses a unique phospho-peptide binding domain called PBD (polo-box domain). Typically, a substrate of polo-like kinase is first phosphorylated by another kinase, and then this priming phosphorylation promotes PBD-dependent substrate recognition and phosphorylation of the substrate by polo-like kinase. *B,* mutations in PBD. *Top,* sequence alignment of two segments in PBD from various species, generated by ClustalW2 (63). Highly conserved residues are in *bold*, and residues where missense mutations are introduced are colored. *Hs, Homo sapiens*; *Xl, Xenopus laevis*; *Ce, Caenorhabditis elegans*; *Sp, Schizosaccharomyces pombe*; *Sc, S. cerevisiae. Bottom left,* three budding yeast PBD mutants used in this study, *cdc5-mut1*, *-mut2,* and *-mut3*. Introduced missense mutations are shown. *Bottom right,* space-filling model of human Plk1 PBD bound to phosphopeptide (*red stick*) (Protein Data Bank accession code, 1UMW). Colored residues are those corresponding to the mutation sites of budding yeast *cdc5-mut1*, *-mut2,* and *-mut3*. Data were drawn with Jmol. *C,* chromosome-binding of the mutant Cdc5 proteins measured by ChIP-qPCR. In the used strains, PK-tagged wild-type or mutant Cdc5 (*mut1, mut2,* and *mut3*) was expressed from a CEN-plasmid-borne gene placed under its native promoter, and the endogenous wild-type Cdc5 was controlled by a galactose-inducible promoter. The cells grown in galactose-containing media were arrested in G_1_ phase by α-factor and then cultivated in galactose-free YPD for 30 min to repress Cdc5 expression. Subsequently, the cells were released from the arrest and re-arrested in G_2_/M phase by culturing in YPD containing benomyl for 3 h. The resultant cells were subjected to anti-PK ChIP-qPCR analysis. *none*, cells harboring an empty vector. *Error bars* indicate standard deviations (*n* = 2, technical replications in qPCR measurements). *D,* ChIP-seq analysis of PK-tagged Cdc5 possessing the PBD mutations, mut1, mut2 or mut3. The experiment was performed similarly to *C*. The *y* axis represents a fold enrichment ratio or ChIP/input value (60). The peaks highlighted in *red* and *orange* indicate statistically significant enrichment with ChIP/input value of more than 2 and 1.5, respectively. Regions shaded in *green* correspond to the hyper-chippable regions (30). *E,* genome-wide correlation between wild-type *CDC5* and *cdc5-mut1* ChIP-seq results. ChIP/input values at each 1-kb bin of the genome (excluding centromeric surrounding regions (±10 kb) and hyper-chippable regions) were plotted. Bins with ChIP/input ratios of more than three for wild-type are in *blue*, and the remainder is shown in *gray. Dots* corresponding to sub-telomeric regions (within 10 kb from the chromosome ends) are shown in *black*.

We next addressed how Cdc5 recognizes the chromatin-bound cohesin complex. Polo-like kinase is characterized by the presence of the polo-box domain (PBD), which functions as a phosphoserine/threonine-binding domain and promotes substrate targeting of polo-like kinase (Fig. 2*A*) (8). Structural and biochemical studies indicate that highly conserved Trp-517, His-641, and Lys-643 (the numbering is for budding yeast Cdc5) in PBD have a crucial role in interacting with a phosphopeptide substrate (9, 10). To determine whether chromatin binding of Cdc5 relies on its PBD function, we introduced mutations to several sites in PBD, including these essential residues (Fig. 2*B*). A Cdc5 PBD mutant consisting of three point mutations, W517F, V518A, and L530A (called *cdc5-mut1* hereafter) was shown to abolish the ability of Cdc5 to localize at subcellular targets and to support yeast cell growth (31). Other mutants, *cdc5-mut2* and *cdc5-mut3*, share substitutions at the “pincer” residues His-641 and Lys-643 of PBD, and in humans the corresponding substitutions impaired the capacity of Plk1 PBD to bind to phosphorylated ligands *in vitro* (9). Yeast cells expressing PK-tagged Cdc5-mut1, Cdc5-mut2, or Cdc5-mut3 proteins from the *CDC5* native promoter were arrested at G_2_/M phase and subjected to anti-PK ChIP-qPCR analysis. The Cdc5-mut1 and -mut2 proteins showed almost complete reduction in binding to the chromosomal cohesin association sites (Fig. 2*C*). The other mutant protein, Cdc5-mut3 also revealed decreased binding, although the degree of decrease is about 30–50%. ChIP-seq analysis of Cdc5-mut1, -mut2, and -mut3 proteins indicated that the dissociation of the PBD-deficient Cdc5 proteins was universally observed throughout the entire genome (Fig. 2, *D* and *E*). Almost all the genome sites with which wild-type Cdc5 is associated (shown as *blue dots* in Fig. 2*E*) exhibited greatly reduced binding of Cdc5-mut1 mutant protein. In conclusion, these data demonstrate that Cdc5 employs PBD to associate with the chromosome-bound cohesin complex.

##### PBD Targets a Middle Part of Scc1 Cohesin Subunit

**FIGURE 3.**
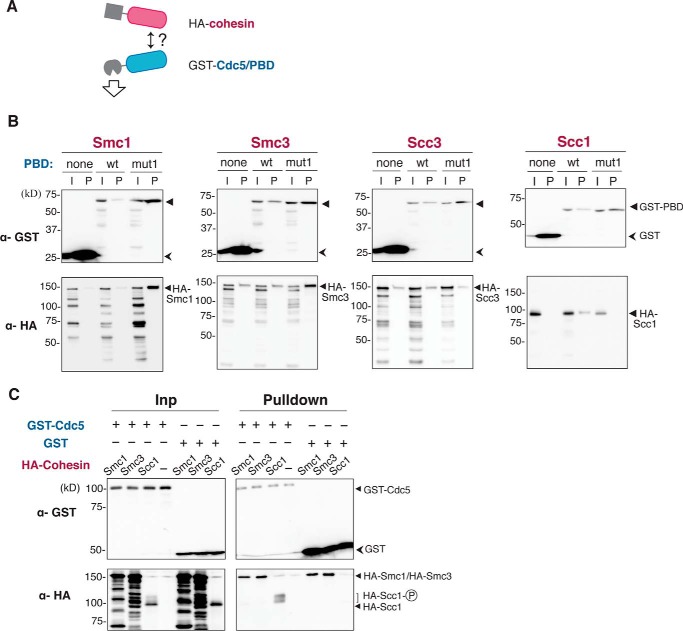
**Cohesin subunit Scc1 is co-purified with PBD of Cdc5.** *A,* experimental scheme to dissect physical interaction of intact Cdc5 or isolated PBD with cohesin. GST-fused Cdc5/PBD and a cohesin subunit (Smc1/Smc3/Scc3/Scc1) tagged with HA epitope were co-overexpressed in yeast cells. GST-Cdc5/PBD was affinity-purified by GST pulldown, and co-purification of the cohesin subunit was examined by Western blotting. *B,* interaction between PBD and each cohesin subunit. Images of Western blotting by anti-GST (α-GST) and anti-HA (α-HA) antibodies are shown. *I*, input; *P*, pulled down material; *none*, GST tag only; *wt*, GST fused with wild-type PBD; *mut1*, GST fused with PBD carrying the *mut1* mutation. *C,* interaction between the intact Cdc5 and a cohesin subunit. HA-tagged Smc1, Smc3, or Scc1 was co-overexpressed with either GST-Cdc5 or GST tag only in yeast cells, and GST pulldown assay was performed for each cell lysate. *Inp*, input.

Because it is now evident that Cdc5 recruitment to chromatin-bound cohesin is dependent on its PBD, we sought for which cohesin subunit mediates the interaction with PBD. For this purpose, either of the cohesin subunits (Smc1, Smc3, Scc1, or Scc3) fused with the HA epitope was overexpressed simultaneously with GST-fused PBD in yeast cells. GST-PBD was affinity-purified from the cell lysate by glutathione (GSH)-Sepharose beads, and co-purified cohesin subunits were visualized by Western blotting detecting HA epitope (Fig. 3*A*). As shown in Fig. 3*B*, the Scc1 subunit was co-purified with GST-PBD. Importantly, co-precipitation was not seen in control assays, where GST tag only (unfused GST) or GST-PBD with the *mut1* mutation was expressed instead of the wild-type GST-PBD. Although the expression level of wild-type and mutant GST-PBD in yeast cells was almost identical, the wild-type GST-PBD was consistently recovered with less efficiency than the mutant one, due to an unknown reason. Nevertheless, significantly more HA-Scc1 was co-purified with the wild-type GST-PBD than with the mutant, indicating high affinity of Scc1 for functional PBD. In contrast to the Scc1 subunit, Smc1, Smc3 and Scc3 showed no co-purification specific to wild-type PBD. For Smc1 and Smc3, a significant amount of protein was co-purified with the mutant GST-PBD, implying that the observed co-precipitation was independent of the integrity of PBD and was presumably due to nonspecific interactions. The specific interaction between Scc1 and functional PBD was verified in the experiment where the full-length Cdc5 fused with GST was overexpressed instead of GST-PBD. In this experiment, slowly migrating forms of Scc1 appeared upon expression of GST-Cdc5, and remarkably, these presumably hyper-phosphorylated Scc1 species showed higher affinity to Cdc5 (Fig. 3*C*), which is consistent with the notion that PBD binds to a phosphorylated protein.

**FIGURE 4.**
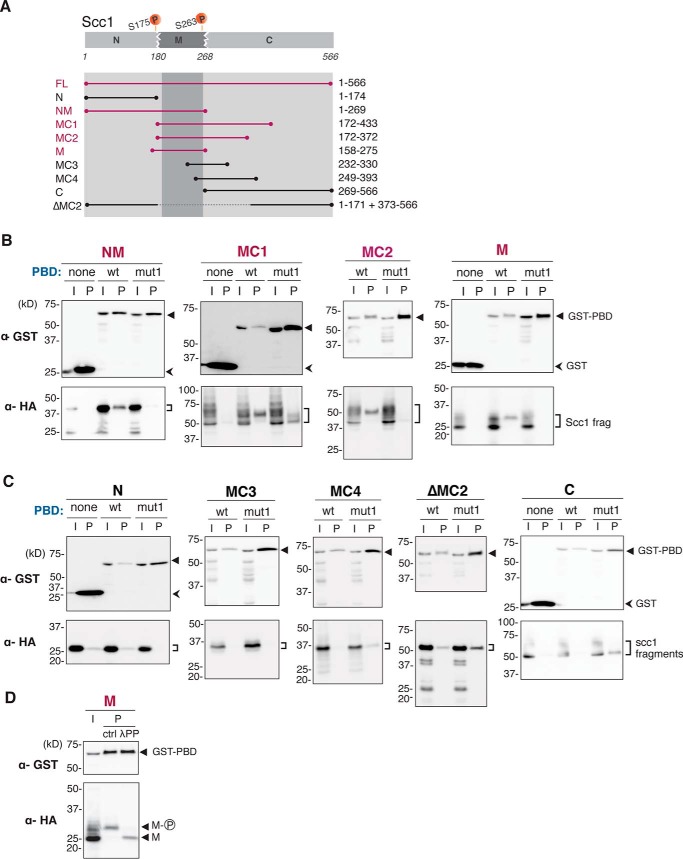
**PBD of Cdc5 interacts with a middle part of Scc1.** *A,* summary of used Scc1 fragments. Scc1 can be divided into three parts, *N*, *M*, and *C*, at two separase-cleavage sites at Arg-180 and Arg-268, and each fragment is named after the part(s) of the fragment covers. Fragments shown in *magenta* demonstrated interaction with PBD (*B*). Ser-175 and Ser-263 are the known phosphorylation sites by Cdc5. *B* and *C,* interaction between PBD and Scc1 fragments were analyzed by GST pulldown assay. Images of Western blotting by anti-GST (α-GST) and anti-HA (α-HA) antibodies are shown. *I*, input; *P*, pulled down material; *none,* GST tag only; *wt*, GST fused with wild-type PBD; *mut1*, GST fused with PBD carrying the *mut1* mutation. *D,* phosphatase treatment of Scc1 M fragment bound to PBD. The M fragment co-purified with PBD (*P*) was treated by λPP and compared with untreated material (*ctrl*).

To exactly define which part of Scc1 interacts with PBD, several Scc1 truncated fragments fused with HA epitope were generated (Fig. 4*A*). Each of the Scc1 fragments was co-overexpressed with GST-PBD in yeast cells, and its association with PBD was examined using GST pulldown assay. Scc1 can be divided into three parts (N-terminal, middle, and C-terminal parts, respectively) by the two separase-dependent cleavage sites (13). As shown in Fig. 4*B*, the fragment extending from the N-terminal to middle part of Scc1 (amino acids 1–269; named NM) revealed specific co-purification with wild-type GST-PBD. Similarly, two other fragments sharing the entire middle part (M) (MC2, amino acids 172–372; M, amino acids 158–275) showed interaction with wild-type GST-PBD but not with GST only nor with mutated GST-PBD. Multiple bands seen in these fragments were likely to be due to phosphorylation of the fragments (see below). Fragment MC1 (amino acids 172–433) was co-purified both with wild-type and mutated GST-PBD. As described above, however, the amount of the purified wild-type GST-PBD protein was significantly smaller than that of mutant GST-PBD, indicating that MC1 also exhibited higher affinity to wild-type PBD. In contrast, the fragments that do not span the middle part (*i.e.* N, MC3, MC4, C, and ΔMC2 in Fig. 4*A*) were not co-purified specifically with wild-type GST-PBD (Fig. 4*C*). We therefore conclude that the middle part is an essential region for Scc1 to be recognized by PBD. It should be noted that the two Cdc5 phosphorylation sites for efficient cleavage, Ser-175 and Ser-263, locate within or very close to the middle region (21).

Some of the PBD-interacting Scc1 fragments showed multiple bands in Western blot analysis, and slowly migrating forms were selectively co-purified with GST-PBD (MC1, MC2, and M in Fig. 4*B*). We therefore examined whether the slow migration was due to phosphorylation of the fragment. The middle part (M) of Scc1 was co-overexpressed with GST-PBD, and the slowly migrating form of the M fragment was purified by GSH-Sepharose. Treatment of the obtained material with λ protein phosphatase (λPP) converted the slowly migrating form to a faster migrating form, indicating that slow migration was due to phosphorylation (Fig. 4*D*). Collectively, our data suggest that PBD recognizes phosphorylated forms of the Scc1 M fragment.

##### PBD Binding Requires Multiple Phosphorylation in the Scc1 Middle Part

**FIGURE 5.**
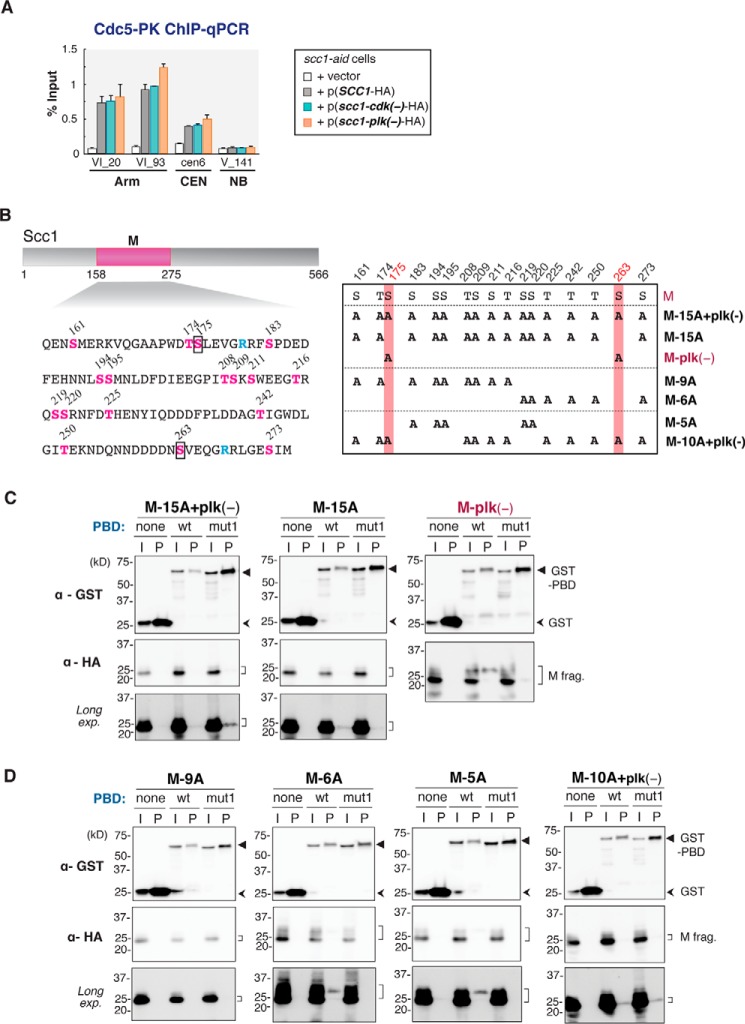
**Dissection of priming phosphorylation sites within the Scc1 middle part.** *A,* Cdc5-PK ChIP-qPCR analysis of Scc1 mutated in potential Cdk phosphorylation sites or Cdc5 phosphorylation sites. *CDC5*-PK *scc1-aid* cells expressing either wild-type Scc1-HA (*SCC1*-HA), Scc1-HA with alanine substitutions at Cdk phosphorylation consensus sites (T140A, S183A, T354A, and T476A) (*scc1-cdk*(−)-HA), or Scc1-HA with alanine substitutions at the Cdc5 phosphorylation sites (S175A and S263A) (*scc1-plk*(−)*-*HA) were arrested at G_1_ phase by α-factor, and the endogenous Scc1 was degraded by addition of 1 mm IAA. Then, cells were released from the arrest and re-arrested at G_2_/M phase by culturing in media containing benomyl and IAA for 2 h. Chromosomal binding of Cdc5-PK was measured by ChIP-qPCR. *Error bars* indicate standard deviations (*n* = 2, technical replications in qPCR measurements). *B,* introduced alanine substitution mutations within the Scc1 M fragment. *Left,* protein sequence of the M fragment. All serine and threonine (*S* or *T*) residues in the fragment are shown in *magenta*. Two arginine (*R*) residues in *cyan* are the separase-induced cleavage sites. *Right,* sites of alanine substitutions (*A*) in the generated mutant Scc1 M fragments are shown. Fragments shown in *magenta* demonstrated interaction with PBD. The known phosphorylation sites by Cdc5, Ser-175, and Ser-263, are marked by *black rectangles* (*left*), or shaded in *red* (*right*). *C* and *D,* GST pulldown assay of Scc1 M fragments possessing the indicated alanine substitutions. *I,* input; *P,* pulled down material; *none,* GST tag only; *wt*, GST fused with wild-type PBD; *mut1*, GST fused with PBD carrying the *mut1* mutation. *Long exp*. indicates long exposure images.

**FIGURE 6.**
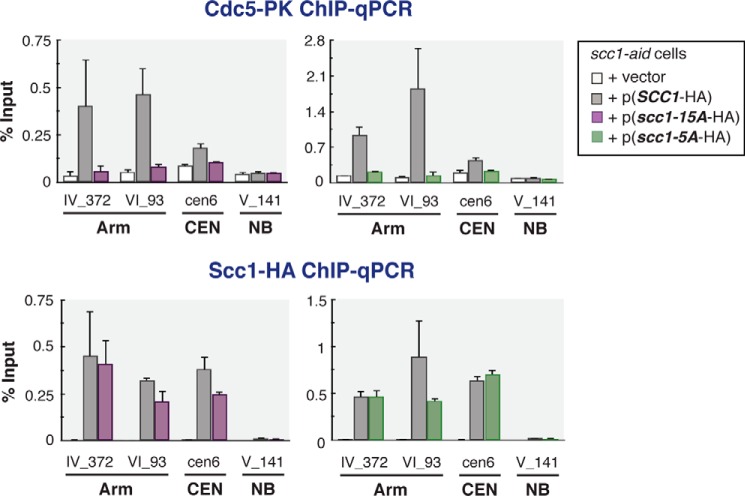
**Chromosomal localization of Cdc5 in alanine-substituted Scc1.** Reduction of Cdc5 chromosome binding in G_2_/M phase by the alanine substitutions. Chromosomal binding of Cdc5-PK as well as wild-type/mutated Scc1-HA was measured by ChIP-qPCR. *CDC5*-PK *scc1-aid* cells expressing from a plasmid either of wild-type Scc1-HA (*SCC1*-HA), Scc1-HA with the 15A substitutions (*scc1-15A*-HA), or Scc1-HA with the 5A substitutions (*scc1–5A*-HA) were cultured and subjected for ChIP-qPCR analysis similarly to cells in Fig. 5*A. Error bars* indicate standard deviations (*n* = 2, technical replications in qPCR measurements).

As described already, PBD targets phosphoserine- or phosphothreonine-containing peptide, and this phosphorylation to be targeted by PBD is called priming phosphorylation. It is shown that the priming phosphorylation is catalyzed by Cdk kinase in many cases. For instance, Cdk1/cyclin B phosphorylates a structural protein of the Golgi apparatus, Grasp65, in its C-terminal domain at four consensus sites, which in turn become recognition sites by Plk1 PBD (32, 33). Because Scc1 contains the minimal Cdk phosphorylation consensus motif (Ser/Thr-Pro) at four sites (Thr-140, Ser-183, Thr-354, and Thr-476), we first substituted all the corresponding Ser/Thr residues to alanine (*scc1-cdk*(−)) and examined whether this substitution impairs Cdc5 binding to Scc1. ChIP-qPCR analysis revealed that *scc1-cdk*(−) mutation did not affect the binding of Cdc5 (Fig. 5*A*), indicating that phosphorylation at other site(s) has an important role in PBD recruitment. To determine the essential priming phosphorylation site(s) in the Scc1 middle region, we generated a set of mutated M fragments, in which all Ser/Thr residues within this region were systematically substituted with alanine (Fig. 5*B*). There are 17 Ser/Thr residues in this region. It was shown that among them, Ser-175 and Ser-263 were phosphorylated by Cdc5 kinase itself, and these phosphorylations promote efficient cleavage of Scc1 by separase (21). We found that alanine substitution of the two Cdc5 phosphorylation sites (M-plk(−)) neither resulted in disappearance of the slowly migrating forms on SDS-PAGE nor abolished the ability of the M fragment to be recognized by PBD in GST pulldown assay (Fig. 5*C*). Consistently, chromosomal binding of Cdc5 measured by ChIP-qPCR was not changed in cells expressing Scc1-plk(−) mutant protein (Fig. 5*A*). In contrast, alanine substitution of all the other 15 Ser/Thr residues in the M fragment (M-15A) impaired PBD binding to the fragment (Fig. 5*C*). ChIP-qPCR analysis also revealed that the same substitution in the full-length Scc1 caused almost complete dissociation of Cdc5 from the cohesin-binding sites without affecting chromosome binding of cohesin itself (Fig. 6). Notably, the hyper-phosphorylated form of the M fragment that was co-purified with GST-PBD (Fig. 4*D*) was no longer detected for the M-15A mutant fragment. These data strongly suggest that the priming phosphorylation essential for PBD recruitment to Scc1 has occurred at some of these 15 Ser/Thr residues (*i.e.* Ser-161, Thr-174, Ser-183, Ser-194, Ser-195, Thr-208, Ser-209, Ser-211, Thr-216, Ser-219, Ser-220, Thr-225, Thr-242, Thr-250, and Ser-273).

To narrow down further the priming phosphorylation sites, we tested other Scc1 M fragments with different sets of alanine substitutions (Fig. 5*B*). Among them, M-5A mutant fragment, in which Ser-183, Ser-194, Ser-195, Ser-219, and Ser-220 are substituted to alanine, diminished the interaction with PBD almost completely (Fig. 5*D*). ChIP-qPCR analysis confirmed Cdc5 dissociation from the chromosomal cohesin sites *in vivo* by the same 5A substitutions (Fig. 6). The five serine residues mutated in M-5A include two sets of two consecutive serine residues. It is noticeable that phosphorylation at the second serine of these sequences produces the serine-phosphoserine (Ser-Ser(P)) motif, which was shown to bind preferentially to PBD (8). However, we also found that the five serine residues mutated in the M-5A were not sufficient to recruit PBD; M-10A+plk(−) mutant fragment, in which the five serines are intact and all the other Ser/Thr residues in the fragment are replaced with alanines (Fig. 5*B*), showed very little interaction with PBD, like M-5A (Fig. 5*D*). These results suggest that multisite phosphorylation of the Scc1 middle region is required for recruiting PBD to cohesin.

##### Scc1 Priming Phosphorylation Is Dispensable for Cell Viability

**FIGURE 7.**
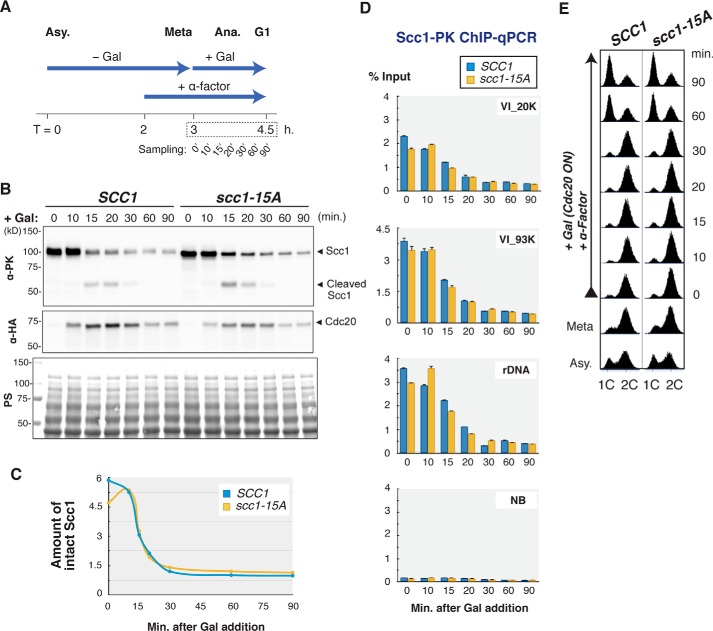
**Priming phosphorylation was not required for timely Scc1 cleavage in anaphase.** *A,* experimental scheme. P*GAL-3HA-CDC20* strains harboring a chromosomally integrated *SCC1-*PK or *scc1-15A-*PK were arrested at metaphase by culturing in galactose-free medium for 3 h and then released from the arrest by galactose-induced Cdc20 expression. The resultant cells progressing through anaphase synchronously were collected at 0, 10, 15, 20, 30, 60, and 90 min after the release. Re-synthesis of Scc1 in the next cell cycle was suppressed by α-factor addition. The harvested cells were subjected to Western blotting and anti-PK ChIP-qPCR analysis. *B,* visualization of Scc1 cleavage and degradation in anaphase by Western blotting. Whole cell lysate of the synchronized cells were prepared and analyzed by anti-PK (α-PK, for Scc1) and anti-HA (α-HA, for Cdc20) antibodies. Ponceau S-stained image (*PS*) is shown to monitor the amount of loaded proteins. *C,* quantification of the full-length Scc1 amount (arbitrary units, *y* axis) revealed by Western blotting (*B*). *D,* quantification of chromosome-associated Scc1 amount by anti-PK ChIP-qPCR. Chromosome binding was measured at cohesin-binding sites on chromosome arms (*VI_20K* and *VI_93K*) and in rDNA loci (*rDNA*) as well as at the site where cohesin shows no binding (*NB*). *Error bars* indicate standard deviations (*n* = 2, technical replications in qPCR measurements). *E,* verification of cell cycle synchronization by flow cytometric analysis.

It was shown that Cdc5 polo-like kinase phosphorylates the Scc1 subunit at Ser-175 and Ser-263, and this phosphorylation promotes efficient Scc1 cleavage by separase in anaphase (21). In Cdc5-depleted cells, the cleavage of Scc1 in anaphase proceeded more slowly than wild-type cells. If the priming phosphorylation of Scc1 discovered in this work is required for Cdc5 to induce the cleavage-enhancing phosphorylation at Ser-175 and Ser-263, one could expect an inefficient cleavage of Scc1 in anaphase when the priming phosphorylation is impaired by alanine substitutions of the 15 Ser/Thr sites in the middle part. To test whether this is the case, we measured the levels of Scc1 and its cleavage product in cells progressing through anaphase synchronously (Fig. 7, *A* and *E*). In wild-type (*SCC1*) cells, a cleaved form of Scc1 became visible by 15 min after induction of Cdc20, an activator of the anaphase-promoting complex, and the amount of the intact Scc1 protein was reduced to less than 20% of the initial level by 30 min (Fig. 7, *B* and *C*). Because the cleaved form is targeted for degradation by the N-end rule pathway (34), the cleaved product is not stoichiometric with the loss of Scc1. In *scc1-15A* mutant cells, where the *scc1* gene has 15 alanine substitutions at the same sites as mutated in the M-15A fragment, the timing of the cleaved form appearance and the decline of the full-length Scc1 was almost indistinguishable from those in wild type (Fig. 7, *B* and *C*). We also monitored dissociation of cohesin from chromosomes by ChIP-qPCR. The amount of chromosome-bound wild-type Scc1 as well as Scc1-15A mutant protein decreased very similarly when cells proceed through anaphase (Fig. 7*D*). These results indicate that the priming phosphorylation in the Scc1 middle part plays little, if any, role in Scc1 anaphase cleavage in wild-type cells.

**FIGURE 8.**
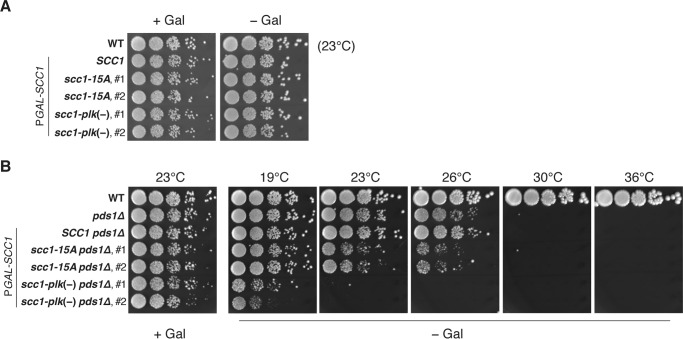
**Genetic interaction between *scc1-15A* and *pds1*Δ mutants.** *A,* wild-type (*SCC1*), *scc1-15A,* and *scc1-plk*(−) strains that harbor a chromosomally integrated *SCC1* gene expressed from galactose-inducible promoter (P*GAL-SCC1*) were analyzed by spotting assay (7-fold serial dilution). Two independent clones were included for each mutant. The cells were grown at 23 °C for 4 days on galactose-containing YPGal (+*Gal*) and galactose-free YPD (−*Gal*) plates, where the ectopic wild-type *SCC1* was expressed and repressed, respectively. *WT* is wild-type yeast without P*GAL-SCC1* gene. *B,* cells possessing *pds1* gene deletion (*pds1*Δ) were analyzed similarly to *A*. Galactose-free (− *Gal*) plates were incubated at various temperatures. The plates were incubated for 6 days (19 °C), 4 days (23 and 26 °C), or 3 days (30 and 36 °C).

We next utilized genetic approach to assess physiological relevance of the Scc1 priming phosphorylation. Alexandru *et al.* (21) reported that loss of the cleavage-enhancing phosphorylation by S175A and S263A substitutions (*i.e. scc1-plk*(−) mutation) resulted in chromosome segregation defects and eventual cell death when combined with *pds1* gene deletion (*pds1*Δ), whereas *scc1-plk*(−) mutation on its own did not hinder cell proliferation. This genetic interaction is consistent with the notion that Pds1 and the phosphorylation of Scc1 at Ser-175/Ser-263 by Cdc5 function redundantly in promoting timely and efficient cleavage of Scc1 in anaphase (21). First, we confirmed this synthetic growth defect phenotype. The *scc1-plk*(−) mutation enhanced temperature sensitivity of the *pds1*Δ mutant (Fig. 8*B*) but did not affect cell growth of wild-type cells (Fig. 8*A*) when expression of an ectopic wild-type *SCC1* was repressed by transferring to galactose-free medium. At 23 °C, the *scc1-plk*(−) *pds1*Δ double mutant formed no colonies, although the *pds1*Δ single mutant grew as well as wild type. We then examined genetic interaction between *scc1-15A* and *pds1*Δ mutants. The *scc1-15A* mutant showed no growth defect on its own (Fig. 8*A*). Unlike *scc1-plk*(−) mutant, *scc1-15A* mutant revealed only a modest synthetic growth defect when combined with *pds1*Δ mutation (Fig. 8*B*). At 23 and 26 °C, the double mutant was able to form colonies, although the number of visible colonies was reduced ∼10-fold compared with the *pds1*Δ single mutant. This result implies that the Scc1 priming phosphorylation plays only a marginal role in enhancing anaphase Scc1 cleavage in cells lacking securin, where Scc1 phosphorylation by Cdc5 is indispensable.

##### Scc1 Priming Phosphorylation Has a Role in DNA Damage Response

**FIGURE 9.**
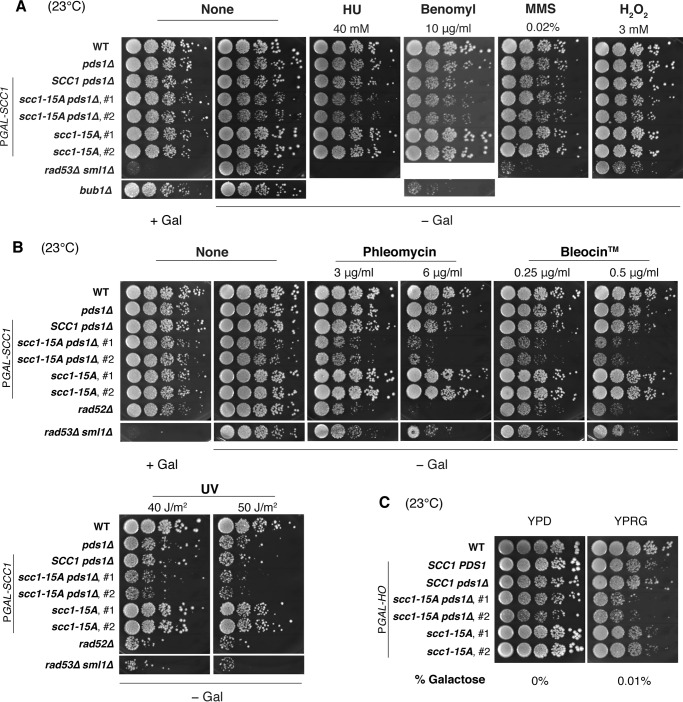
**Sensitivity of *scc1-15A pds1*Δ double mutant to DNA-damaging agents.** *A* and *B,* serial dilution assay of the same strains used in Fig. 8 on plates containing various drugs or after ultraviolet irradiation. *A* and *B,* cells were grown on plates without drugs (*None*) or plates containing HU (40 mm), benomyl (10 μg/ml), MMS (0.02%), hydrogen peroxide (H_2_O_2_, 3 mm), phleomycin (3 or 6 μg/ml), or Bleocin^TM^ (0.25 or 0.5 μg/ml). Alternatively, the cells spotted on a plate were irradiated by 254 nm of UV light at a dose of 40 or 50 J/m^2^ before incubation. P*GAL-SCC1* indicates the presence of an ectopic copy of wild-type *SCC1* expressed from a galactose-inducible promoter, expression of which is repressed in galactose-free (−*Gal*) condition. *rad53*Δ *sml1*Δ and *rad52*Δ strains were used as positive controls for HU, MMS, phleomycin, Bleocin^TM^, and UV sensitivity, whereas *bub1*Δ used as a positive control for benomyl sensitivity. *C,* serial dilution assay of cells with indicated genotypes under the condition that chromosome cleavage was induced by HO endonuclease expression. Except for WT, the used cells contained *GAL1*-inducible *HO* gene (P*GAL1-HO*) and an ectopic HO target site on chromosome V, and the DNA cleavage was promoted by weak induction of HO (0.01% galactose).

Cohesin is known to be involved not only in sister chromatid cohesion, but also in the DNA double-stranded break repair process (35, 36). We therefore investigated whether the priming phosphorylation of Scc1 has a role in cells challenged by DNA-damaging agents or other drugs that affect cell cycle progression or cell proliferation. Growth of *scc1-15A* and *scc1-15A pds1*Δ cells on plates containing drugs was measured by spotting serial dilutions of liquid cultures. None of methylmethane sulfonate (MMS, alkylating agent inducing DNA damage), hydroxyurea (HU, DNA replication inhibitor), benomyl (microtubule-destabilizing reagent), or H_2_O_2_ (an oxidant) caused a growth defect specific to *scc1-15A* or *scc1-15A pds1*Δ strains (Fig. 9*A*). Interestingly, we found that growth of *scc1-15A pds1*Δ cells was severely impaired in the presence of phleomycin, a bleomycin-related antibiotic, whereas wild-type and *scc1-15A* cells grew normally, and *pds1*Δ cells showed only modest growth defect under the same conditions (3 and 6 μg/ml phleomycin) (Fig. 9*B*). The *scc1-15A pds1*Δ cells also exhibited a growth defect on a plate containing Bleocin^TM^, a formulation containing bleomycin (Fig. 9*B*), indicating that the double mutant was generally sensitive to this family of antibiotics.

Phleomycin and bleomycin is known to induce double-stranded breaks (DSB) in DNA (37, 38). We therefore examined whether *scc1-15A pds1*Δ cells show a growth defect in the presence of DSB caused by HO endonuclease, a site-specific double-stranded endonuclease. HO endonuclease was expressed from galactose-inducible promoter in wild-type, *pds1*Δ, *scc1-15A,* and *scc1-15A pds1*Δ cells in which an HO endonuclease cleavage site was integrated into chromosome V. HO induction by 0.05% or a higher concentration of galactose impeded colony formation in spotting assay, regardless of genotype (data not shown). At a lower expression level of HO induced by 0.01% galactose, however, the *scc1-15A pds1*Δ double mutant specifically showed growth defect (Fig. 9*C*). This result is consistent with the notion that *scc1-15A pds1*Δ is defective in cellular response to DSB.

In addition to DSB, we observed that *scc1-15A pds1*Δ cells were also sensitive to ultraviolet (UV) irradiation, which forms a thymine dimer lesion in DNA. When irradiated with 254 nm UV light at 50 J/m^2^, the *pds1*Δ mutant showed reduced viability, compared with wild type (Fig. 9*B*). The *scc1-15A pds1*Δ double mutant exhibited further reduced viability, implying that the Scc1 priming phosphorylation and Pds1 synergistically function in response to UV-induced DNA lesions. Taken together, the priming phosphorylation of Scc1 seems to have an important role in responding to DNA lesions, including DSB.

##### Cdc5 Is Preferentially Associated with Cohesin Engaged in Cohesion

**FIGURE 10.**
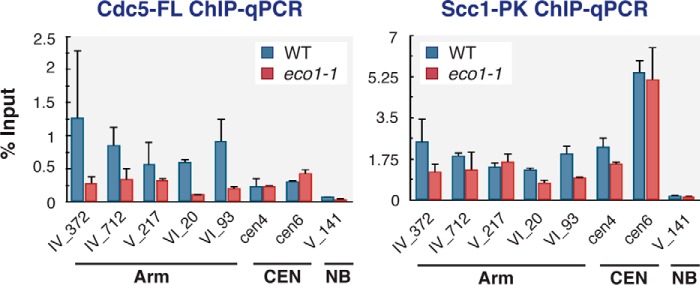
**Cdc5 association with cohesin on chromosome arms was attenuated in *eco1-1* mutant.** Wild-type (*WT*) and *eco1-1* strains possessing FLAG-tagged *CDC5* and PK-tagged *SCC1* genes were cultured at 23 °C and arrested in G_1_ phase by α-factor. To inactivate Eco1, cells were shifted to restrictive temperature (35 °C) for 30 min while arresting at G_1_. Then the cells were released into benomyl-containing media at 35 °C for 2 h. The resultant G_2_/M phase cells were subjected to anti-FLAG and anti-PK ChIP-qPCR analysis. The used qPCR loci correspond to cohesin localization sites on chromosome arms (*Arm*) or at the centromeres (*CEN*), except no binding (*NB*) site where no cohesin was seen. *Error bars* indicate standard deviations (*n* = 2, technical replications in qPCR measurements).

We finally addressed why Cdc5 showed preferential binding to cohesin on chromosome arms and not to that around the centromeres. It is known that chromosome binding of cohesin is more dynamic around the centromeres, implying that the majority of centromeric cohesin is not engaged in sister chromatid cohesion (39, 40). Cdc5 may exhibit higher affinity to cohesin mediating cohesion, and we tested this hypothesis utilizing the *eco1-1* mutant. Eco1 acetylates cohesin in S phase, thereby enabling it to bind chromosomes stably and to establish sister chromatid cohesion (41). In the *eco1-1* mutant, we found that the amount of chromosome-bound Scc1-PK in G_2_/M phase cells was reduced slightly (by ∼30% on average) on chromosome arms (Fig. 10). This reduction may reflect presumably dynamic chromosome binding of cohesin in this mutant. In contrast, the amount of chromosome-bound Cdc5-FL was more significantly reduced in *eco1-1* cells (by ∼70% on average). Interestingly, Cdc5 binding to the centromeric loci was not affected by *eco1-1* mutation. These results support the above-mentioned hypothesis that Cdc5 exhibits higher affinity to cohesin engaged in sister chromatid cohesion, which may explain the disfavored association of Cdc5 with centromeric cohesin.

## Discussion

In this study, we discovered that Cdc5 polo kinase is associated with cohesin complex localized on budding yeast chromosome arms prior to anaphase onset. The PBD mutations impairing the binding of PBD to phosphopeptide abolished the Cdc5 association with cohesin, and PBD was preferentially bound to phosphorylated Scc1 fragments *in vitro*. These results suggest that the observed Cdc5-Scc1 association reflects PBD-dependent targeting of Cdc5 kinase to its substrate. This notion is consistent with the previous reports that Scc1 is phosphorylated by Cdc5 in mitotic yeast cells (5, 21, 22). In cohesin-depleted cells, chromosomal binding of Cdc5 revealed by ChIP-seq almost completely disappeared. Hence, cohesin is likely to be the only major target of PBD-dependent Cdc5 pre-deposition among the proteins bound to budding yeast G_2_/M phase chromosomes. It is also of note that the Cdc5-mut1 protein was localized specifically at sub-telomeric regions along chromosome arms (Fig. 2*E*). Cdc5 may interact with a telomeric protein in a PBD-independent manner.

Chromosomal association of Cdc5 was greatly diminished in a mutant of Eco1, which acetylates cohesin specifically during S phase and promotes establishment of sister chromatid cohesion. Hornig and Uhlmann (22) reported that Cdc5 preferentially phosphorylates chromatin-bound Scc1, and our data deepen the understanding by revealing that cohesion establishment, rather than loading onto chromosomes, may convert cohesin to a more preferred target for Cdc5. The preferred association of Cdc5 to acetylated cohesin presumably promotes rapid and efficient dissolution of chromatid cohesion at anaphase onset, thereby contributing to accuracy of chromosome segregation. It remains to be understood how acetylation could result in preferential recognition by Cdc5.

GST pulldown assay using truncated and alanine-substituted Scc1 fragments demonstrated that an ∼120-amino acid-long Scc1 middle part binds to Cdc5 PBD in a phosphorylation-dependent manner. The same region contains the cleavage sites by separase, and the sites of Cdc5 kinase phosphorylation for efficient cleavage (13, 21) thus form a central target of mitotic regulations of cohesin. Phosphorylation for PBD recruitment, or priming phosphorylation, is typically catalyzed by Cdk. In the case of Scc1, however, kinase(s) other than Cdk are most likely to be involved in the priming phosphorylation, because alanine substitution at all the potential Cdk target sites in Scc1 did not prevent Cdc5 recruitment to cohesin. Another example of the known priming kinases for PBD recruitment is Plk itself (42). The priming phosphorylation by Plk is known as self-priming phosphorylation, exemplified by cases of microtubule-associated PRC1 and mitotic kinesin MKlp2, and generally occurred in substrates that are activated during post-metaphase stages (43, 44), when Cdk activity declines. Indeed, besides the well studied Cdc5 target sites for cleavage (Ser-175, Ser-263), Scc1 was shown to be phosphorylated by human Plk1 *in vitro* at several additional sites, and three of them (Ser-183, Ser-194, and Ser-273) are located within the middle part (21). In fission yeast, Rad21 was shown to be phosphorylated by a DNA-damage checkpoint kinase ATR in UV-irradiated cells (45). Various kinases may function as priming kinases in a cellular context-dependent manner. It remains to be elucidated which kinase(s) catalyze the priming phosphorylation of Scc1.

The middle part of Scc1 possesses 15 Ser/Thr as potential priming phosphorylation sites. Systematic alanine substitution revealed a set of five Ser/Thr (Ser-183, Ser-194, Ser-195, Ser-219, and Ser-220), which include two of the abovementioned *in vitro* Plk target sites, as a minimum requirement for PBD recruitment. We, however, failed to identify a single residue whose phosphorylation is sufficient for PBD binding, because PBD binding was severely impaired by both alanine substitutions of two non-overlapping sets of Ser/Thr (M-9A and M-6A, Fig. 5*D*). Notably, we also observed that among several phosphorylated forms of Scc1 M fragments, only a molecular species with relatively slow mobility on SDS-PAGE showed high affinity to PBD (Fig. 4*D*). These results strongly suggest that multisite phosphorylation in the middle region is required for promoting efficient PBD binding to cohesin. Multisite phosphorylation is a common feature of many protein kinase substrates. In previous studies of such an example, a Cdk inhibitor, Sic1, multisite phosphorylation is proposed to enable an ultrasensitive switch-like response to a gradually increasing kinase activity (46, 47). Scc1 phosphorylation at multiple sites may also constitute a molecular switch that contributes to all-or-none activation of Scc1 cleavage reaction.

We found that wild-type Scc1 and Scc1 deficient in the priming phosphorylation Scc1-15A were cleaved with similar kinetics in anaphase, despite the fact that Cdc5 was almost completely dissociated from chromosomal cohesin in *scc1-15A* cells. This result is not unexpected, because Cdc5-dependent phosphorylation and the securin Pds1 are known to play a redundant role in promoting efficient and timely cleavage of Scc1 in anaphase (21). Consistently, *scc1-plk*(−) mutant, which lacks the target sites of Cdc5 phosphorylation for efficient cleavage, showed synthetic lethality with a *pds1*Δ mutant at 23 °C or higher temperatures (Fig. 8*B*). We therefore addressed how *scc1-15A* mutant cells grow when combined with *pds1* deletion, and we revealed that they showed only moderate sickness. This result indicates that the priming phosphorylation is not an absolute requirement for Ser-175 and Ser-263 phosphorylation of Scc1 for anaphase cleavage. Based on the cellular phenotype of the Cdc5 PBD mutant, PBD-substrate interaction, but not phosphorylation by Cdc5, is thought to be largely dispensable for mitotic progression in budding yeast (11). In this study, however, the requirement of priming phosphorylation for Scc1 cleavage was not rigorously addressed because the cells possessed the *PDS1* gene, and therefore, phosphorylation by Cdc5 itself was dispensable for Scc1 cleavage. Thus, this study assessed fully for the first time how physiologically important the priming phosphorylation is for Scc1 cleavage in the cells where phosphorylation by Cdc5 is vital for the cleavage.

Strikingly, the *scc1-15A pds1*Δ double mutant exhibited elevated sensitivity to genotoxic agents phleomycin and bleomycin, demonstrating that the priming phosphorylation of Scc1 would play a more significant role under certain circumstances. DSB is a type of DNA lesions caused by phleomycin/bleomycin. Cohesin is known to be required for post-replicative DSB repair and to be actively loaded onto the genome regions surrounding DSB sites (36, 48). Once DSB repair is completed, the excessively loaded cohesin probably has to be removed before and/or upon chromosome segregation, and it requires accelerated Scc1 cleavage reaction, for which the priming phosphorylation may be required. Notably, Cdc5 is needed for yeast cells to adapt to the permanent presence of DSB and to re-enter the cell cycle (49). One of the Cdc5 substrates in adaptation might be a cohesin subunit Scc1. The *scc1-15A pds1*Δ double mutant showed no elevated sensitivity to other genotoxic agents, MMS and HU. These agents also produce DSB in cells, but the DSBs are thought to arise during S phase in a replication-dependent manner (50, 51). The difference of the timing and mechanism of DSB occurrence may account for the observed sensitivity to phleomycin/bleomycin but not to MMS and HU. In fission yeast, *rad21* mutant cells containing alanine substitutions at the ATR target sites exhibited increased sensitivity to a different genotoxic stress, ultraviolet irradiation, when combined with separase or securin mutation (45). Consistently, budding yeast *scc1-15A pds1*Δ cells also showed elevated sensitivity to UV light, compared with wild-type and *pds1*Δ cells. The Scc1 priming phosphorylation is likely to function in responding to UV-induced DNA lesions in addition to DSB. It remains to be explored how the Scc1 priming phosphorylation functions in cellular response to these genotoxic environments.

## Experimental Procedures

### 

#### 

##### Yeast Strains and Plasmids

*S. cerevisiae* strains used in this study are wild-type BY4741 and its derivatives (except the strains used in Figs. 1*F*, 9*C,* and 10, which are W303 derivatives) and are listed in Table 1. Epitope tagging of a chromosomal gene was performed by one-step PCR-based strategy (52). For *aid* module tagging, pMK43 plasmid (29) was used as a PCR template. Replacements of an endogenous promoter with a galactose-inducible *GAL1* promoter as well as gene deletion were also conducted by one-step PCR-based strategy using template plasmids described previously (53). Among the alanine-substituted mutants of the Scc1 middle fragment, M-15A, M-15A+plk(−), and M-10A+plk(−) were generated by gene synthesis. The other Scc1 mutants were created by site-directed mutagenesis based on overlap extension PCR (54), using wild-type and the above-mentioned synthesized *scc1* fragments as templates. Site-directed mutagenesis of Cdc5 PBD was also conducted by overlap extension PCR. DNA sequences of the created fragments cloned onto plasmids were found as expected by Sanger sequencing in all cases. For expression of the PBD-deficient Cdc5 proteins, the mutagenized genes placed under the *CDC5* native promoter were cloned into a CEN plasmid YCplac111, and the resultant plasmids were transformed into yeast cells. For expression of full-length Scc1 with alanine substitutions, the mutagenized *scc1* genes that are placed under its native promoter and fused with HA epitopes were cloned into a CEN plasmid YCplac22, and the resultant plasmids were transformed into yeast cells. For chromosomal integration of the mutated *scc1* genes, the mutated genes placed under its native promoter were cloned into the backbone of pFA6a-3HA-TRP1 plasmid, together with PK epitopes and *His3MX6* marker. Using the resultant plasmids as templates, one-step replacement of the endogenous *SCC1* gene with the PK-tagged mutant *scc1* genes was conducted. Strains used to measure sensitivity to HO endonuclease were created from the parental strain that contains HO gene controlled by *GAL1* promoter and an ectopic HO recognition site integrated on chromosome V (48). All the above-mentioned chromosomal integration/replacements were verified by colony PCR. The introduction of the alanine substitutions into genome was confirmed by Sanger sequencing of the corresponding genome regions.

**TABLE 1:** ***S. cerevisiae* strains used in this study** chr. is chromosome.

Relevant figure	Strain ID	Genotype
Fig. 1, *A* and *B*	SP18	*MAT*a *his3*Δ*1 leu2*Δ*0 met15*Δ*0 ura3*Δ*0 trp1*::*hisG SCC1-9PK*:*TRP1 CDC5-6FL*:*kanMX6*
Fig. 1, *A, D,* and *E*	SP91	*MAT*a *his3*Δ*1 leu2*Δ*0 met15*Δ*0 ura3*Δ*0 trp1*::*hisG aur1*::*AUR1-C,*P*ADH1-AtTIR1-9myc scc1-aid*:*kanMX6 CDC5-9PK*:*His3MX6*
Fig. 1, *A, D,* and *E*	SP90	*MAT*a *his3*Δ*1 leu2*Δ*0 met15*Δ*0 ura3*Δ*0 trp1*::*hisG aur1*::*AUR1-C,*P*ADH1-AtTIR1-9myc smc3-aid*:*kanMX6 CDC5-PK*:*His3MX6*
Fig. 1*F*	SP27*^a^*	*MAT*a *ade2-1 trp1-1 can1-100 leu2-3,12 his3-11,15 ura3 CDC5-FL*:*kanMX6*
Fig. 1*F*	SP54*^a^*	*MAT*a *ade2-1 trp1-1 can1-100 leu2-3,12 his3-11,15 ura3 smc3-42 CDC5-6FL*:*kanMX6*
Fig. 1*G*	SP47	*MAT*a *his3*Δ*1 leu2*Δ*0 met15*Δ*0 ura3*Δ*0 trp1*::*hisG SMC3-9PK*:*TRP1*
Fig. 1*G*	SP57	*MAT*a *his3*Δ*1 leu2*Δ*0 met15*Δ*0 ura3*Δ*0 trp1*::*hisG SMC3-9PK*:*TRP1* P*GAL1-3HA-CDC5*:*His3MX6*
Fig. 2*C*	SP125	*MAT*a *his3*Δ*1 leu2*Δ*0 met15*Δ*0 ura3*Δ*0 trp1*::*hisG* P*GAL1-3HA-CDC5*:*His3MX6* [p(*LEU2, CEN4)*]
Fig. 2, *C-E*	SP126	*MAT*a *his3*Δ*1 leu2*Δ*0 met15*Δ*0 ura3*Δ*0 trp1*::*hisG* P*GAL1-3HA-CDC5*:*His3MX6* [p(*CDC5-9PK, LEU2, CEN4)*]
Fig. 2, *C-E*	SP127	*MAT*a *his3*Δ*1 leu2*Δ*0 met15*Δ*0 ura3*Δ*0 trp1*::*hisG* P*GAL1-3HA-CDC5*:*His3MX6* [p(*cdc5-W517F,V518A,L530A-9PK, LEU2, CEN4)*]
Fig. 2, *C* and *D*	SP276	*MAT*a *his3*Δ*1 leu2*Δ*0 met15*Δ*0 ura3*Δ*0 trp1*::*hisG* P*GAL1-3HA-CDC5*:*His3MX6* [p(*cdc5-W517F,H641A,K643M-9PK, LEU2, CEN4)*]
Fig. 2, *C* and *D*	SP275	*MAT*a *his3*Δ*1 leu2*Δ*0 met15*Δ*0 ura3*Δ*0 trp1*::*hisG* P*GAL1-3HA-CDC5*:*His3MX6* [p(*cdc5-H641A,K643M-9PK, LEU2, CEN4)*]
Fig. 3, *B* and *C*	SKY001*^b^*	*MAT*a *his3*Δ*1 leu2*Δ*0 met15*Δ*0 ura3*Δ*0 trp1*::*hisG*
Fig. 4, *B-D*		
Fig. 5, *C* and *D*		
Fig. 5*A*	SP96	*MAT*a *his3*Δ*1 leu2*Δ*0 met15*Δ*0 ura3*Δ*0 trp1*::*hisG aur1*::*AUR1-C,*P*ADH1-AtTIR1–9myc scc1-aid*:*kanMX6*
Fig. 6		*CDC5-9PK*: *His3MX6* [p(*TRP1, CEN4)*]
Fig. 5*A*	SP97	*MAT*a *his3*Δ*1 leu2*Δ*0 met15*Δ*0 ura3*Δ*0 trp1*::*hisG aur1*::*AUR1-C,*P*ADH1-AtTIR1–9myc scc1-aid*:*kanMX6*
Fig. 6		*CDC5-9PK*: *His3MX6 [p*(*SCC1-3HA, TRP1, CEN4)]*
Fig. 5*A*	SP104	*MAT*a *his3*Δ*1 leu2*Δ*0 met15*Δ*0 ura3*Δ*0 trp1*::*hisG aur1*::*AUR1-C,*P*ADH1-AtTIR1–9myc scc1-aid*:*kanMX6 CDC5-9PK*:*His3MX6* [p(*scc1-T140A,S183A,T354A,T476A-3HA, TRP1, CEN4)*]
Fig. 5*A*	SP110	*MAT*a *his3*Δ*1 leu2*Δ*0 met15*Δ*0 ura3*Δ*0 trp1*::*hisG aur1*::*AUR1-C,*P*ADH1-AtTIR1–9myc scc1-aid*:*kanMX6 CDC5-9PK*:*His3MX6* [p(*scc1-S175A,S263A-3HA, TRP1, CEN4)*]
Fig. 6	SP392	*MAT*a *his3*Δ*1 leu2*Δ*0 met15*Δ*0 ura3*Δ*0 trp1*::*hisG aur1*::*AUR1-C,*P*ADH1-AtTIR1–9myc scc1-aid*:*kanMX6 CDC5-9PK*:*His3MX6* [p(*scc1-S161A,T174A,S183A,S194A,S195A,T208A,S209A,S211A,T216A,S219A,S220A,T225A, T242A,T250A,S273A-3HA, TRP1, CEN4)*]
Fig. 6	SP394	*MAT*a *his3*Δ*1 leu2*Δ*0 met15*Δ*0 ura3*Δ*0 trp1*::*hisG aur1*::*AUR1-C,*P*ADH1-AtTIR1–9myc scc1-aid*:*kanMX6 CDC5-9PK*:*His3MX6* [p(*scc1-S183A,S194A,S195A,S219A,S220A-3HA, TRP1, CEN4)*]
Fig. 7. *B-E*	SP425	*MAT*a *his3*Δ*1 leu2*Δ*0 met15*Δ*0 ura3*Δ*0 trp1*::*hisG* P*GAL1-3HA-CDC20*:*TRP1 CDC5–6FL*:*kanMX6 SCC1–9PK*:*His3MX6*
Fig. 7, *B-E*	SP426	*MAT*a *his3*Δ*1 leu2*Δ*0 met15*Δ*0 ura3*Δ*0 trp1*::*hisG* P*GAL1-3HA-CDC20*:*TRP1 CDC5–6FL*:*kanMX6 scc1-S161A,T174A,S183A,S194A,S195A,T208A,S209A,S211A,T216A,S219A,S220A,T225A,T242A,T250A, S273A-9PK*:*His3MX6*
Fig. 8 *A* and *B*	SP13	*MAT*a *his3*Δ*1 leu2*Δ*0 met15*Δ*0 ura3*Δ*0 trp1*::*hisG CDC5–6FL*:*kanMX6*
Fig. 9, *A* and *B*		
Fig. 8*A*	SP493	*MAT*a *his3*Δ*1 leu2*Δ*0 met15*Δ*0 ura3*Δ*0 trp1*::*hisG CDC5–6FL*:*kanMX6 aur1*::*AUR1-C,*P*GAL1-SCC1-3HA SCC1–9PK*:*His3MX6*
Fig. 8*A*	SP494-3	*MAT*a *his3*Δ*1 leu2*Δ*0 met15*Δ*0 ura3*Δ*0 trp1*::*hisG CDC5–6FL*:*kanMX6 aur1*::*AUR1-C,*P*GAL1-SCC1-3HA*
Fig. 9, *A* and *B*	SP494-5	*scc1-S161A,T174A,S183A,S194A,S195A,T208A,S209A,S211A,T216A,S219A,S220A,T225A,T242A,T250A,S273A-9PK*: *His3MX6*
Fig. 8*A*	SP495-8	*MAT*a *his3*Δ*1 leu2*Δ*0 met15*Δ*0 ura3*Δ*0 trp1*::*hisG CDC5–6FL*:*kanMX6 aur1*::*AUR1-C,*P*GAL1-SCC1-3HA*
	SP495-9	*scc1-S175A,S263A-9PK*:*His3MX6*
Fig. 8*B*	SP435	*MAT*a *his3*Δ*1 leu2*Δ*0 met15*Δ*0 ura3*Δ*0 trp1*::*hisG CDC5–6FL*:*kanMX6 pds1*Δ::*LEU2*
Fig. 9, *A* and *B*		
Fig. 8*B*	SP496	*MAT*a *his3*Δ*1 leu2*Δ*0 met15*Δ*0 ura3*Δ*0 trp1*::*hisG CDC5–6FL*:*kanMX6 aur1*::*AUR1-C,*P*GAL1-SCC1-3HA SCC1–9PK*: *His3MX6 pds1*Δ::*LEU2*
Fig. 9, *A* and *B*		
Fig. 8*B*	SP499a-1	*MAT*a *his3*Δ*1 leu2*Δ*0 met15*Δ*0 ura3*Δ*0 trp1*::*hisG CDC5–6FL*:*kanMX6 aur1*::*AUR1-C,*P*GAL1-SCC1-3HA*
Fig. 9, *A* and *B*	SP499b-2	*scc1-S161A,T174A,S183A,S194A,S195A,T208A,S209A,S211A,T216A,S219A,S220A,T225A,T242A,T250A,S273A-9PK*: *His3MX6 pds1*Δ::*LEU2*
Fig. 8*B*	SP502a-1	*MAT*a *his3*Δ*1 leu2*Δ*0 met15*Δ*0 ura3*Δ*0 trp1*::*hisG CDC5–6FL*:*kanMX6 aur1*::*AUR1-C,*P*GAL1-SCC1-3HA*
	SP502b-1	*scc1-S175A,S263A-9PK*:*His3MX6 pds1*Δ::*LEU2*
Fig. 9*A,B*	SKY050*^b^*	*MAT*a *his3*Δ*1 leu2*Δ*0 met15*Δ*0 ura3*Δ*0 trp1*::*hisG rad53*Δ::*URA3 sml1*Δ::*LEU2*
Fig. 9*A*	SP526	*MAT*a *his3*Δ*1 leu2*Δ*0 met15*Δ*0 ura3*Δ*0 trp1*::*hisG bub1*Δ::*LEU2*
Fig. 9*B*	SP547	*MAT*a *his3*Δ*1 leu2*Δ*0 met15*Δ*0 ura3*Δ*0 trp1*::*hisG rad52*Δ::*LEU2*
Fig. 9*C*	SP26*^a^*	*MAT*a *ade2-1 trp1-1 can1-100 leu2-3,12 his3-11,15 ura3*
Fig. 9*C*	SP530*^a^*	*MAT*a *ade3*::P*GAL-HO ade2-1 trp1-1 can1-100 leu2-3,112 his3-11,15 ura3 GAL psi+ RAD5*; *HO recognition site* (*chr.V_541 kb)*
Fig. 9*C*	SP534-3*^a^*	*MAT*a *ade3*::P*GAL-HO ade2-1 trp1-1 can1-100 leu2-3,112 his3-11,15 ura3 GAL psi+ RAD5*; *HO recognition site* (*chr.V_541 kb) SCC1-3HA*:*TRP1 pds1*Δ::*LEU2*
Fig. 9*C*	SP535a-2*^a^*	*MAT*a *ade3*::P*GAL-HO ade2-1 trp1-1 can1-100 leu2-3,112 his3-11,15 ura3 GAL psi+ RAD5*; *HO recognition site*
	SP535b-5*^a^*	(*chr.V_541 kb) scc1-S161A,T174A,S183A,S194A,S195A,T208A,S209A,S211A,T216A,S219A,S220A,T225A,T242A,*
		*T250A,S273A-3HA*:*TRP1 pds1*Δ::*LEU2*
Fig. 9*C*	SP533-13*^a^*	*MAT*a *ade3*::P*GAL-HO ade2-1 trp1-1 can1-100 leu2-3,112 his3-11,15 ura3 GAL psi+ RAD5*; *HO recognition site*
	SP533-24*^a^*	(*chr.V_541 kb) scc1-S161A,T174A,S183A,S194A,S195A,T208A,S209A,S211A,T216A,S219A,S220A,T225A,T242A,*
		*T250A,S273A-3HA*:*TRP1*
Fig. 10	SP28*^a^*	*MAT*a *ade2-1 trp1-1 can1-100 leu2-3,12 his3-11,15 ura3 CDC5–6FL*:*kanMX6 SCC1–9PK*:*HIS3MX6*
Fig. 10	SP24*^a^*	*MAT*a *ade2-1 trp1-1 can1-100 leu2-3,12 his3-11,15 ura3 eco1-1 CDC5–6FL*:*kanMX6 SCC1–9PK*:*HIS3MX6*

*^a^* W303-1a derivatives were used. The parental strains are described in Michaelis *et al.* (64) (*smc3-42*), Ström *et al.* (48) (Gal-HO), and Tóth *et al.* (65) (*eco1-1*).

*^b^* Data are from Katou *et al.* (57).

For GST pulldown assay in yeast cells, the DNA fragment encoding the intact Cdc5 or PBD of Cdc5 (amino acids 357–705) was cloned into pEG(KT) multicopy plasmid (55), which contains the *GAL1-10* inducible promoter and GST tag. To overexpress HA-tagged Scc1, its truncated fragments, or other cohesin subunits, the corresponding DNA fragments were cloned together with the *GAL1* promoter and 3HA epitope into a YEplac112 multicopy plasmid. Cells harboring the resultant plasmids were used for the assay. The parental pEG(KT) plasmid (empty vector) was used as a negative control to express the GST tag only (unfused GST).

##### Cell Culture

Yeasts were cultured in complete YPD (56) medium at 23 °C unless otherwise mentioned. Cells were synchronized in G_1_ phase by culturing in medium containing 2 μm α-factor (peptide synthesized by Sigma) for 2.5 h. Release from the G_1_ arrest was induced by adding 150 μg/ml Pronase (Calbiochem) to the medium. To be arrested in G_2_/M phase, cells were cultured in medium containing 80 μg/ml benomyl (Sigma) for 2.5 h. For preparation of G_2_/M phase-arrested cells lacking a cohesin subunit Scc1 or Smc3 by use of *aid* system, cells were arrested in G_1_ phase by cultivating in α-factor-containing medium for 2 h, and then selective degradation of the aid-fused Scc1/Smc3 was induced by adding 1 mm IAA (Sigma; dissolved in ethanol at 500 mm) and culturing for another 1 h. Subsequently, the cells were released from G_1_ arrest and re-arrested in G_2_/M phase by changing the medium to fresh YPD containing Pronase, benomyl, and IAA and culturing for additional 2 h. In preparation of cells progressing through anaphase synchronously, cells with P*GAL-3HA-CDC20*, where the endogenous Cdc20 is N-terminally tagged with 3HA epitope and controlled by the *GAL1* promoter, were grown in YPR (YPD containing 2% raffinose instead of glucose) containing 2% galactose and then transferred to and incubated in YPR for 3 h to promote Cdc20 depletion and metaphase arrest. Proper arrest was confirmed by accumulation of large-budded cells under microscopy. Finally, the cells were released synchronously from the arrest by adding 2% galactose to the medium.

For selection and maintenance of transformants with auxotrophic marker(s), synthetic complete (SC) medium lacking appropriate nutrient(s) (56) was used. In functional analysis of PBD-deficient Cdc5 proteins, the mutant Cdc5 proteins were expressed from plasmid-borne genes in P*GAL1–3HA-CDC5* cells, where the endogenous Cdc5 is N-terminally tagged with 3HA epitope and controlled by the *GAL1* promoter. For preparation of cells arrested in G_2_/M phase and lacking the endogenous Cdc5, the cells were first cultured in SGal-L (SC containing 2% galactose instead of glucose and lacking leucine) and arrested in G_1_ phase by addition of 2 μm α-factor for 3 h. Then the cells were transferred to and incubated in YPD containing α-factor for 30 min to promote Cdc5 depletion and finally re-arrested in G_2_/M phase by adding Pronase and benomyl to the medium and culturing for an additional 2 h. In analysis of Scc1-15A and Scc1-5A proteins in G_2_/M phase-arrested *scc1-aid* strains, the cells were first grown in SC medium lacking tryptophan and arrested in G_1_ phase by adding 2 μm α-factor for 2 h. Then the cells were transferred to and incubated in YPD containing IAA and α-factor for 1 h to promote Scc1-aid depletion and finally re-arrested in G_2_/M phase by adding Pronase and benomyl to the medium and culturing for an additional 2 h. For GST pulldown assay, cells with overexpression plasmids were grown in SRaf-UraTrp (SC containing 2% raffinose instead of glucose, lacking uracil and tryptophan) at 30 °C and then cultured in YPR for 2 h. Subsequently protein expression and G_2_/M phase arrest were promoted by including 2% galactose and benomyl in the medium for 3 h. Strains harboring the galactose-inducible *SCC1* gene (P*GAL1-SCC1*) at *AUR1* locus were created, maintained, and grown in YPRG (YPR with 2% galactose) medium before spotting assay.

##### Serial Dilution Spotting Assay

Yeasts were inoculated in either YPRG (for P*GAL1-SCC1* harboring strains) or YPD and allowed to grow overnight at 23 °C. Medium was washed out, and the cells were resuspended in 10% glycerol before counting by a hemocytometer. The initial density of cells was adjusted to 2.4 × 10^6^ cells/ml, and 7-fold dilution was performed sequentially four times. 5 μl of each diluted cell suspension was spotted onto YPRG or YPD solid media (the number of dispensed cells were 12,005 and five cells in the first and fifth spots, respectively). The following chemical agents were used in plate assay to challenge yeast cell cycle and/or cell growth: 3 or 6 μg/ml phleomycin (Sigma), 0.25 or 0.5 μg/ml Bleocin^TM^ (Calbiochem), 0.02% MMS (Sigma), 40 mm HU (Tokyo Chemical Industry), 10 μg/ml benomyl (Sigma), 3 mm H_2_O_2_ (Wako). These agents were added to melted warm YPD agar before pouring into sterile Petri dishes. To induce DNA lesions by UV radiation, the cells spotted on solid media were exposed to UV-C light (at 254 nm wavelengths) generated by UV cross-linkers (Ultra-Violet Products Ltd.) at a dose of 40 or 50 J/m^2^. Strains harboring P*GAL1-HO* were pre-cultured in YPR and spotted onto YPD or YPR with 0.01% galactose. After spotting, the plates were incubated at the indicated temperature until colonies of wild-type control were formed.

##### Flow Cytometry

Cell cycle synchronization was monitored by flow cytometric DNA quantification as described (56). The analysis was conducted on a FACSCalibur or Accuri C6 flow cytometer (BD Biosciences).

##### Antibodies

Monoclonal anti-PK (SV5-Pk1 clone, AbD Serotec, catalog MCA1360), monoclonal anti-FLAG (M2 clone, Sigma, catalog F1804), monoclonal anti-GST (5A7 clone, Wako, catalog 013-21851), and polyclonal anti-aid (or IAA17, Cosmo Bio, catalog APC004A) antibodies were used for ChIP and/or Western blotting. For the HA epitope, monoclonal anti-HA antibody clones 12CA5 (Roche Applied Science, catalog 11-583-816-001) and 16B12 (BioLegend, catalog 901503) were used for Western blot and ChIP, respectively. Antibody dilution in Western blot analysis was 1:1,000 for all antibodies. Peroxidase-conjugated antibodies against mouse and rabbit IgG (Jackson ImmunoResearch, catalog 115-035-062 and 111-035-144, respectively) were used as secondary antibodies in Western blot analysis.

##### Chromatin Immunoprecipitation (ChIP)

ChIP analysis was performed as described previously (57). For anti-HA ChIP analysis, bridging antibody for mouse IgG (Active Motif, catalog 53017) was used to increase the amount of anti-HA antibody captured on protein A-conjugated magnetic beads.

##### Quantitative PCR Analysis of ChIP-purified DNA

ChIP-purified DNA was quantified using KAPA SYBR Fast qPCR kit (KAPA Biosystems) and real time PCR systems 7500 and StepOnePlus (Life Technologies, Inc.) as per the manufacturer's instructions. Both input and ChIP DNA, or DNA before and after ChIP, were measured in duplicate. The level of enrichment in ChIP was calculated as a ratio of the amount of ChIP DNA over that of input, or *C_m_/I_m_*, where *C_m_* is the mean of the ChIP DNA measurements, and *I_m_* is the mean of the input DNA measurements. The primers used in qPCR are listed in Table 2.

**TABLE 2:** **Primer pairs used for ChIP-qPCR assay** chr is chromosome.

Target site name	Forward sequence (5′–3′)	Reverse sequence (5′–3′)	Chromosome position
IV_372	TTATAGCGGAACTACGTTCGCCTTCT	AGGACTCCAATAATCCAGCCTTGCAT	chr IV, 372,807- 372,896
cen4	AGCATCGTATACAGGAAGTGCCATGA	TAGTTTCTGTGCTGTGCGTGATGTTC	chr IV, 448,118–448,214
IV_712	TTCCACCAGGTCTAGGAGTAGCACTGT	TCGTCGGACCTTATTGAGGAGTTGTC	chr IV, 712,311–712,415
V_217	GCCAATCGTCACAATCGGGTAGTAGT	GACCTGTTAATGGTCACAGAAGGTTGAGA	chr V: 217,056–217,164
VI_141 (NB)	GCTGGGTCTCACGATCCATATCAG	TCTTGTTTCGGTGAGTTGGACAGATC	chr V: 140,946–141,091
VI_20	GCTGCCAGTGTTGCTGTTGCTG	GGAGCCTGGGGTGGTCCAATTGC	chr VI: 20,874–21,083
VI_93	CAAGACAAGTCGTTTCCGCTCTCAAC	CTTTATCGAAACCAATCCTGCTGCTAG	chr VI: 93,325–93,550
cen6	GCGAAAAAGGCTCCGAAGAAGTTTG	TGGCGCTAACTCCCTTGTCTGTTC	chr VI: 147,359–147,599
rDNA	ACATACTAAATCTCTTCCCGTCATTATCG	GACAAATGGATGGTGGCAGGCATAG	chr XII: 459,260–459,375, chr XII: 468,397–468,512

##### ChIP-seq Analysis

High-throughput sequencing and analysis of ChIP-purified DNA was conducted, as described previously (58), with several minor modifications. Sequencing was carried out using SOLiD 5500 (Applied Biosystems) and HiSeq 2000 (Illumina) systems. Input and ChIP DNA were processed and sequenced following the manufacturers' instructions. More than five million of single-end 50- or 65-bp reads were generated for each sample. The obtained reads were mapped to the *S. cerevisiae* reference genome using Bowtie (version 1.1.0) (59), with “—best” option. The mapping results of ChIP and the corresponding input DNA were fed into ChIP-seq analysis tool package DROMPA (version 2.5.3) (60) with “PEAK CALL_E” and “-sm500” options. This manipulation compressed reads sharing the same 5′ end into a single read to minimize PCR bias and then generated a genome-wide list of ChIP/input values, each of which is a ratio of the number of ChIP sequence reads mapped at a specific 100-bp genome segment to the number of input sequence reads mapped at the same genome site, and smoothened with a 500-bp size window. The ChIP/input value reflects the degree of enrichment in the ChIP procedure. DROMPA also visualized the generated ChIP/input value list as a genome-wide ChIP-seq profile with results of statistical test for enrichment. Genome-wide correlation between two ChIP-seq results was analyzed and plotted using R. Genome sequence and gene annotation were obtained from SGD and NCBI (www.ncbi.nlm.nih.gov), respectively. Sequencing and mapping statistics are summarized in Table 3.

**TABLE 3:** **Sequencing and mapping statistics for ChIP-seq data**

	Sample description	Platform	Reference	Total number of reads	No. and percentage (in parentheses) of mapped reads	
ChIP	Cdc5-6FL	SOLiD 5500	BY4741 genome	6,039,906	4,037,964	(79.2%)
	Scc1-9PK	SOLiD 5500	BY4741 genome	5,401,671	3,788,354	(81.5%)
	Cdc5-9PK, scc1-aid (+IAA)	Hiseq 2000	BY4741 genome	5,461,067	4,024,849	(76.1%)
	Cdc5-9PK, scc1-aid (+vehicle)	Hiseq 2000	BY4741 genome	6,124,349	3,675,456	(65.6%)
	Cdc5-9PK, smc3-aid (+IAA)	Hiseq 2000	BY4741 genome	6,141,267	4,745,439	(80.2%)
	Cdc5-9PK, smc3-aid (+vehicle)	Hiseq 2000	BY4741 genome	4,416,222	3,447,091	(80.2%)
	Cdc5-9PK (mut1)	Hiseq 2000	BY4741 genome	7,680,113	5,764,250	(78.2%)
	Cdc5-9PK (mut2)	Hiseq 2000	BY4741 genome	7,389,253	5,908,232	(81.7%)
	Cdc5-9PK (mut3)	Hiseq 2000	BY4741 genome	7,172,884	5,675,683	(81.2%)
Input	Cdc5–6FL Scc1-9PK, input DNA	SOLiD 5500	BY4741 genome	5,411,149	4,142,239	(87.9%)

##### Protein Analysis

Yeast protein extract was prepared from trichloroacetic acid (TCA)-treated yeast cells as described (61). For time course analysis of Scc1 cleavage in anaphase, yeast cells in 10 ml of culture were harvested by filtration, dipped into an ice-cold STOP buffer (150 mm NaCl, 1 mm NaN_3_, 50 mm NaF, 10 mm EDTA, pH 8.0), and stored on ice until all samples were harvested. Then, the cells were washed once with 0.5 ml of *in situ* buffer (50 mm Hepes-KOH, pH 7.5, 100 mm KCl, 2.5 mm MgCl_2_, 0.4 m sorbitol), resuspended in 40 μl of extraction buffer (50 mm Hepes-KOH, pH 7.5, 100 mm KCl, 2.5 mm MgCl_2_, 1 mm DTT, 1× cOmplete protease inhibitor mixture (Roche Applied Science), 1 mm phenylmethylsulfonyl fluoride (PMSF)), mixed with 120 μl of 2× SDS-PAGE sample buffer containing 0.5-mm diameter glass beads (Sigma), and boiled at 95 °C for 5 min with continuous mixing. Subsequently, the cells were broken by 4 min of vigorous shaking (2,700 rpm) in a Multi-beads Shocker (Yasui Kikai), and the collected crude lysate was boiled for another 5 min at 95 °C. The lysate was clarified by centrifugation at 14,000 rpm for 10 min, and the supernatant was subjected to Western blot analysis.

SDS-PAGE and Western blotting were performed as described previously (62). Total protein on the blotting membrane was visualized by Ponceau S staining. Western blot image was acquired by ImageQuant LAS 4000 (GE Healthcare). For signal intensity quantification, band intensity was quantified using ImageQuant TL software (GE Healthcare) and normalized to total protein loading revealed by Ponceau S staining.

##### GST Pulldown Assay

The 40-ml culture of G_2_/M phase-arrested cells co-overexpressing GST-fused PBD (or Cdc5) and HA-tagged Scc1 fragments (or intact cohesin subunit) was harvested and washed once with ice-cold PBS, before flash freezing with liquid N_2_ and storage at −80 °C. Cells expressing mutant PBD fused with GST or unfused GST, instead of GST-PBD, were used as a negative control. The following procedures were performed on ice or at 4 °C. Lysis buffer consisting of 40 mm Hepes-KOH, pH 7.4, 140 mm NaCl, 10% glycerol, 0.1% Triton X-100, 1 mm dithiothreitol, 0.5 mm PMSF, 1× cOmplete protease inhibitor mixture (Roche Applied Science), 1× Protease inhibitor mixture for fungal and yeast extracts (Sigma), 1× PhosSTOP Phosphatase inhibitor mixture (Roche Applied Science) was used for lysate preparation and preparation/wash of affinity beads. The frozen cell pellet was thawed and lysed in 500 μl of Lysis buffer containing 0.5-mm diameter glass beads (Sigma) with 6 min of vigorous shaking (2,700 rpm on a Multibeads Shocker, Yasui Kikai) at 0 °C. The recovered crude lysate was clarified by centrifugation (14,000 rpm 10 min) repeated twice, and then the resultant lysate was mixed with 30 μl of glutathione-Sepharose 4B beads (GSH beads; GE Healthcare) that were pre-equilibrated with Lysis buffer, and incubated for 1 h on a rotor. The beads were subsequently washed three times by spinning down the beads (3,000 rpm 1 min), discarding the supernatant, and resuspending the beads in 800-μl fresh Lysis buffer. Finally, the supernatant was discarded, and the bead-bound proteins were solubilized by boiling with 40 μl of 2× SDS-PAGE sample buffer at 95 °C for 3 min (“pulldown” fraction). An aliquot of the clarified lysate before mixing with GSH beads was used as “input” fraction. To identify a cohesin subunit or Scc1 fragments that are able to bind to functional PBD, the amount of protein under test co-purified with GST-PBD was compared with those co-purified with GST-fused mutant PBD or GST only. The pulldown fractions that correspond to the same number (∼10^8^) of yeast cells at the start were loaded onto a single polyacrylamide gel and subjected to anti-HA Western blotting analysis.

The λPP treatment of the purified proteins was carried out on GSH beads using λPP (New England Biolabs) and NEBuffer for protein metallophosphatase (New England Biolabs). The protein-bound GSH beads after the wash step were subsequently washed twice with NEBuffer for protein metallophosphatase and resuspended in 50 μl of the same buffer. 400 units of λPP was added and incubated at 30 °C for 1 h. After the reaction, the beads were spun down and boiled with 40 μl of 2× SDS-PAGE sample buffer at 95 °C for 3 min.

## Author Contributions

S. P., K. S., and T. S. designed the study. S. P. conducted all the experiments, following the early-phase study of Cdc5-Scc1 co-localization by E. T. M. I. analyzed high-throughput sequencing data. S. P., K. S., and T. S. interpreted the data and prepared the manuscript.

## Footnotes

2 The abbreviations used are:

Plk: polo-like kinase
PBD: polo-box domain
aid: auxin-inducible degron
IAA: indole-3-acetic acid
ChIP-seq: chromatin immunoprecipitation followed by DNA sequencing
λPP: Lambda protein phosphatase
DSB: DNA double-stranded break
qPCR: quantitative PCR
HU: hydroxyurea
MMS: methylmethane sulfonate
ATR: ataxia telangiectasia and Rad3 related.
